# Deletion of an immune evasion gene, *steD*, from a live *Salmonella enterica* serovar Typhimurium vaccine improves vaccine responses in aged mice

**DOI:** 10.3389/fimmu.2024.1376734

**Published:** 2024-06-07

**Authors:** Jessica C. Allen, Shanaliz S. Natta, Shamima Nasrin, Franklin R. Toapanta, Sharon M. Tennant

**Affiliations:** ^1^ Center for Vaccine Development and Global Health, University of Maryland School of Medicine, Baltimore, MD, United States; ^2^ Department of Medicine, University of Maryland School of Medicine, Baltimore, MD, United States

**Keywords:** *Salmonella*, vaccine, live-attenuated, immunosenescence, immune evasion

## Abstract

**Introduction:**

Non-typhoidal *Salmonella* (NTS) generally causes self-limiting gastroenteritis. However, older adults (≥65 years) can experience more severe outcomes from NTS infection. We have previously shown that a live attenuated *S*. Typhimurium vaccine, CVD 1926 (I77 Δ*guaBA* Δ*clpP* Δ*pipA* Δ*htrA*), was immunogenic in adult but not aged mice. Here we describe modification of CVD 1926 through deletion of *steD*, a *Salmonella* effector responsible for host immune escape, which we hypothesized would increase immunogenicity in aged mice.

**Methods:**

Mel Juso and/or mutuDC cells were infected with *S*. Typhimurium I77, CVD 1926, and their respective *steD* mutants, and the MHC-II levels were evaluated. Aged (18-month-old) C57BL/6 mice received two doses of PBS, CVD 1926, or CVD 1926 Δ*steD* perorally (10^9^ CFU) and the number of FliC-specific CD4^+^ T cells were determined. Lastly, aged C57BL/6 mice received three doses of PBS, CVD 1926, or CVD 1926 Δ*steD* perorally (10^9^ CFU) and then were challenged perorally with wild-type *S*. Typhimurium SL1344 (10^8^ CFU). These animals were also evaluated for antibody responses.

**Results:**

MHC-II induction was higher in cells treated with *steD* mutants, compared to their respective parental strains. Compared to PBS-vaccinated mice, CVD 1926 Δ*steD* elicited significantly more FliC-specific CD4^+^ T cells in the Peyer’s Patches. There were no significant differences in FliC-specific CD4^+^ T cells in the Peyer’s patches or spleen of CVD 1926- versus PBS-immunized mice. CVD 1926 and CVD 1926 Δ*steD* induced similar serum and fecal anti-core and O polysaccharide antibody titers after three doses. After two immunizations, the proportion of seroconverters for CVD 1926 Δ*steD* was 83% (10/12) compared to 42% (5/12) for CVD 1926. Compared to PBS-immunized mice, mice immunized with CVD 1926 Δ*steD* had significantly lower *S*. Typhimurium counts in the spleen, cecum, and small intestine upon challenge. In contrast, there were no differences in bacterial loads in the tissues of PBS-vaccinated and CVD 1926-immunized animals.

**Conclusion:**

These data suggest that the *steD* deletion enhanced the immunogenicity of our live attenuated *S*. Typhimurium vaccine. Deletion of immune evasion genes could be a potential strategy to improve the immunogenicity of live attenuated vaccines in older adults.

## 1 Introduction

Non-typhoidal *Salmonella* (NTS) is a leading cause of diarrheal disease worldwide. NTS is a foodborne pathogen that was responsible for 197 million diarrheal episodes and 85,000 deaths globally in 2016 (1). In the United States (U.S.), NTS is the leading cause of foodborne hospitalizations and deaths; the Centers for Disease Control and Prevention (CDC) estimates that there are 1.35 million infections, 26,500 hospitalizations, and 420 deaths due to *Salmonella* infection each year (2). This results in an estimated $400 million in direct medical costs and 32,900 disability adjusted life years (DALYs) in the U.S. each year due to NTS foodborne illness (3). Antibiotics are used to treat severe salmonellosis, however, antimicrobial resistant (AMR) strains are increasingly prevalent and the CDC lists NTS as a serious antibiotic resistant threat that requires prompt and sustained action (4).

Older adults (≥65 years) are especially susceptible to complications of NTS disease, and this is likely due to the gradual decline in immune function with age, termed immunosenescence (5). The Global Burden of Disease Study reported that there were almost 10 million NTS infections amongst people over the age of 70 and 19,056 died of NTS infection in 2016 (1). The case fatality rate is 10 times higher for older adults compared to adults (3.0% versus 0.3%), and seniors account for a large proportion of deaths due to NTS in the U.S. (6, 7). According to data from the Foodborne Diseases Active Surveillance Network (FoodNet), the hospitalization rate is above 50% for individuals over 65, compared to 27% for the total U.S. population (8). The duration of hospitalization care for older adults is 2–3 days longer than for younger patients (7). While healthy individuals experience self-limiting gastroenteritis during infection, older adults are at increased risk for invasive and extraintestinal manifestations such as septicemia, meningitis, acute renal failure, hemolytic uremic syndrome and arrhythmias (7–9). Thus, NTS represents a major public health concern, especially for older individuals, and there is currently no NTS vaccine.

While immunization is the best strategy for mitigating NTS disease, older adults produce suboptimal immune responses following vaccination (5). For example, our group developed a live attenuated *Salmonella enterica* serovar Typhimurium vaccine, CVD 1926 (I77 Δ*guaBA* Δ*clpP* Δ*pipA* Δ*htrA*), and reported that CVD 1926 conferred protection against bacterial burden of the spleen, liver, and small intestine upon challenge in 6–8-week-old mice, but not in 18-month-old animals (equivalent to 65-year-old humans) (10). Eighteen-month-old mice that received CVD 1926 produced lower serum and fecal antibody titers, as well as fewer *Salmonella*-specific CD4^+^ and CD8^+^ T cells in the spleen and Peyer’s Patches as compared to adult mice (10). Age-associated impairments in antigen processing and T cell priming by antigen presenting cells (APC) have been reported, and these deficiencies may explain, at least in part, the weak T cell mediated immunity (T-CMI) elicited by CVD 1926 in aged mice (11).

While our overall goal is to develop a NTS vaccine that would be protective for all age groups, our previous data suggested that CVD 1926 would not be sufficiently immunogenic in older adults (10). Therefore, strategies to improve the immunogenicity of CVD 1926 should be employed. One way in which live vaccines have been modified to enhance immune responses is through deletion of immune evasion genes encoded by the pathogen. Deletion of immune evasion genes from live vaccine strains has been shown to be a successful approach for investigational vaccines against murine influenza A and B virus, mouse cytomegalovirus (MCMV), and gammaherpesvirus 68 (MHV-68; closely related to Epstein–Barr virus [EBV] and Kaposi sarcoma-associated herpesvirus [KSHV]) (12–14). With respect to *Salmonella* vaccines, this approach was explored by Li et al. and Männe et al. (15, 16). Deletion of *sopB*, a *Salmonella* effector involved in inducing host cell invasion and cytoskeletal rearrangement, from a recombinant attenuated *S*. Typhimurium vaccine strain improved the antigen-specific serum IgG and fecal IgA levels and induced more central memory T cells following immunization, as compared to the isogenic *sopB*-positive strain (15). Likewise, the *Salmonella* protein SiiE reduces the number of IgG^+^ plasma cells in the bone marrow and thus leads to lower production of anti-*Salmonella* serum IgG during infection. Inoculation with a *siiE*-deficient attenuated *Salmonella* strain induced higher anti-*Salmonella* serum IgG versus the SiiE-producing strain (16). Both SopB and SiiE execute mechanisms for immune escape and these studies provide proof of concept that removing *Salmonella* immune evasion genes from live vaccine strains can improve immunogenicity. However, whether this approach could be used to boost immune responses sufficiently in the context of aging has not yet been determined.

The goal of the current study was to determine whether deletion of the immune evasion gene, *steD*, from CVD 1926 could enhance immunogenicity and vaccine efficacy in aged mice. As a method of circumventing host adaptive immune responses, *Salmonella* spp. produce the SteD protein, which is a *Salmonella* pathogenicity island 2 (SPI-2) effector that inhibits T cell activation by reducing surface expression of major histocompatibility complex (MHC)-II, CD86 and CD97 (17–19). MHC-II and CD86 are essential for antigen presentation to CD4^+^ T cells and CD97 is a plasma membrane protein that is required for formation of the immunological synapse between dendritic cells (DCs) and T cells (18). Since aged mice displayed weakened antigen processing, reduced T cell priming and lower T-CMI to vaccines, we carefully selected a *Salmonella* gene that targets these immune pathways. Given the pleiotropic effects of SteD (i.e., antigen presentation by DCs and synapse stabilization) and that aged mice exhibit weak T cell responses, we sought to evaluate if deletion of *steD* from our candidate vaccine can overcome these age-related deficiencies and improve CVD 1926 responses in aged mice.

In this study, we aimed to enhance the immunogenicity of CVD 1926 by deleting the *steD* gene. We subsequently evaluated the immunogenicity of CVD 1926 Δ*steD* in 18-month-old C57BL/6 mice. We measured serum IgG and fecal IgA titers, functional antibody responses, *Salmonella*-specific T-cell responses and protection against bacterial colonization of the spleen, cecum, and small intestine, upon challenge with wild-type *S*. Typhimurium.

## 2 Materials and methods

### 2.1 Animals and ethics statement

All animal studies were performed in facilities that are accredited by the Association for Assessment and Accreditation of Laboratory Animal Care. Mice were housed under specific pathogen–free conditions at the University of Maryland School of Medicine, and all the procedures were approved by the University of Maryland Baltimore Institutional Animal Care and Use Committee (protocol no. 0619004 and 0422011). Eighteen-month-old (aged) female and male C57BL/6 mice were acquired from the NIA aged rodent colony or purchased from The Jackson Laboratory.

### 2.2 Bacterial strains and culture conditions

The bacterial strains used in this study are shown in **Table 1**. All *Salmonella* strains were maintained in animal-product-free Hy-Soy (HS) medium (10 g/L Soytone [Teknova, Hollister, CA], 5 g/L Hy-yest [Kerry Bio-Science, Beloit, WI] and 5 g/L sodium chloride [American Bio, Natick, MA]) at 37°C as described (10). When needed, agar (Sigma–Aldrich, St. Louis, MO) was added at 15 g/L. For mutants harboring *ΔguaBA* deletions, medium was supplemented with guanine (0.005% weight/volume final concentration; Sigma-Aldrich). For *S*. Typhimurium SL1344, medium was supplemented with 50 μg/mL streptomycin sulfate (Research Products International, Mt. Prospect, IL). *S*. Typhimurium I77, CVD 1926, and CVD 1926 Δ*steD* were labeled with green fluorescent protein (GFP) by electroporation of plasmid pGEN206, which carries *gfpUV* under expression of the *ompC* promoter (24). Carbenicillin (Corning, Glendale, AZ) or kanamycin (Sigma–Aldrich) were added at a final concentration of 50 µg/mL when necessary.

**Table 1:** *S*. Typhimurium strains and plasmids used in this study.

Strain/Plasmid	Description	Reference
SL1344	Streptomycin-resistant *S*. Typhimurium	(20)
I77	*S*. Typhimurium clinical isolate	(21)
D65	*S*. Typhimurium clinical isolate	(22)
I77 Δ*steD*	*S*. Typhimurium Δ*steD*	This study
CVD 1926	*S*. Typhimurium I77 Δ*guaBA* Δ*clpP* Δ*pipA* Δ*htrA*	(23)
I77 (pGEN206)	GFP-expressing I77; ampicillin-resistant (Amp^R^)	This study
I77 Δ*steD* (pGEN206)	GFP-expressing I77 Δ*steD*; Amp^R^	This study
CVD 1926 Δ*steD*	*S*. Typhimurium I77 Δ*guaBA* Δ*clpP* Δ*pipA* Δ*htrA* Δ*steD*	This study
CVD 1926 (pGEN206)	GFP-expressing CVD 1926; Amp^R^	This study
CVD 1926 Δ*steD* (pGEN206)	GFP-expressing CVD 1926 Δ*steD*; Amp^R^	This study
pGEN206	*ori*101 *gfpuv repA par bla hok*-*sok parA*	(24)
pKD13	Flippase recognition target (FRT)-flanked kanamycin resistant gene (FRT-*aphA*-FRT)	(25)
pKD46	Red recombinase expression plasmid; Amp^R^, λ red genes behind the *araBAD* promoter, temperature-sensitive origin of replication (*ori*)	(25)
pCP20	Flippase vector; Amp^R^, temperature-sensitive *ori*	(25)

### 2.3 Construction of *S*. Typhimurium mutants

*S*. Typhimurium CVD 1926 was previously generated by Higginson et al. (23) derived from *S*. Typhimurium I77, a clinical isolate, and harbors deletions in *guaBA*, *clpP, pipA* and *htrA*. The *steD* gene was deleted from the chromosome of *S*. Typhimurium I77 and CVD 1926, by allelic exchange using the lambda-red system as described (25). All plasmids used in this study are shown in **Table 1**. The entire gene coding region was deleted and was confirmed genotypically by PCR and sequencing using primers that were located at least 500 base pairs outside of the targeted gene.

### 2.4 Cell culture and *in vitro* infection

Mel Juso cells (a human melanoma cell line) were cultured in RPMI 1640 (Thermo Fisher Scientific, Waltham, MA) supplemented with 10% heat-inactivated fetal bovine serum (FBS; Gemini Bioproducts, West Sacramento, CA) and incubated at 37°C in 5% CO_2_. MutuDCs (Applied Biological Materials Inc., Canada), immortalized murine dendritic cells (26), were cultured in Iscove’s Modified Dulbecco’s Medium (IMDM)-Glutamax™ medium (Gibco) supplemented with 10% FBS, 1% of 7.5% Sodium Bicarbonate Solution (Gibco), 50 µM β-mercaptoethanol (Gibco), 10 mM HEPES (Gibco), and 1% Penicillin/Streptomycin Solution and incubated at 37°C in 5% CO_2_.

Mel Juso and mutuDC infections were performed as described (17, 27). Briefly, cells were seeded in a tissue culture-treated 24-well plate (Corning) at 10^5^ cells/well and incubated overnight at 37°C in 5% CO_2_. GFP-expressing *S*. Typhimurium were prepared by performing a 1:100 dilution of an overnight culture in fresh HS medium supplemented with 50 µg/mL carbenicillin and incubating with shaking at 37°C until an OD_600_ of 0.8 was reached. Mel Juso cells were infected at a multiplicity of infection (MOI) of 100:1 (10^7^ CFU) for 30 minutes at 37°C in 5% CO_2_. For mutuDC infection, cells were infected at a MOI of 20:1 (2 x 10^6^ CFU) for 30 minutes at 37°C in 5% CO_2_ with overnight cultures. After incubation with bacteria, cells were washed twice with sterile phosphate buffered saline (PBS; Quality Biologicals, Gaithersburg, MD). RPMI 1640 or IMDM-Glutamax™ containing 100 µg/mL gentamicin was added for 1 hour at 37°C in 5% CO_2_ to remove extracellular bacteria. Cells were then washed twice with sterile PBS and were replaced with RPMI 1640 or IMDM-Glutamax™ for 18 hours at 37°C in 5% CO_2_.

### 2.5 Immunization and challenge

Immunization inoculum was prepared as described in (10). Briefly, aged (18-month-old) C57BL/6 mice (n=12) were immunized by peroral gavage with PBS or 10^9^ CFU of CVD 1926 or CVD 1926 Δ*steD* suspended in 100 μL PBS on days 0 and 28 (to examine T-cell responses) and 0, 28, and 56 (to evaluate protection). For T-cell responses, mice were euthanized 21 days after the last dose, and the spleen and Peyer’s Patches were removed. To evaluate protection, immunized C57BL/6 mice were challenged with 10^8^ CFU of *S*. Typhimurium SL1344 four weeks after the last immunization using the streptomycin mouse model (28), as described in (10). Three days after infection, the spleen, liver, and gastrointestinal tract (cecum and small intestine) were collected and homogenized in sterile PBS. Tissue homogenates were serially diluted and 100 μL was plated on to streptomycin-containing agar to determine the CFU per organ.

### 2.6 Isolation of cells from the spleen and Peyer’s Patches

Single cell suspensions from the spleen and Peyer’s Patches were prepared by mechanical dissociation of organs through a 70-µm-pore nylon filter. For spleens, red blood cells were lysed with 100% Ammonium-Chloride-Potassium (ACK) lysis buffer (Gibco, Grand Island, NY). Prior to mechanical dissociation, whole Peyer’s Patches were immersed in Tissue Dissociation Medium (Stem Cell ***™*** Technologies, Vancouver, Canada) for 15 minutes at 37°C. After dissociation, cells were washed with PBS and then resuspended in complete RPMI 1640 medium (Gibco) supplemented with 10% FBS, 100 U/mL penicillin (Sigma-Aldrich), and 100 µg/mL streptomycin (Sigma-Aldrich).

### 2.7 Flow cytometry

For surface staining of MHC-II on Mel Juso or MutuDC cells, cells were detached from 24-well plates using Accutase^®^ (BioLegend, San Diego, CA) treatment for 25 minutes and were transferred to round bottom polystyrene tubes. Cells were then washed with fluorescence activated cell sorting (FACS) buffer (PBS, 10% FBS, 0.1% sodium azide) and surface stained with antibodies against HLA-DR (PE; L243 monoclonal antibody; BioLegend) or MHC-II (PE; M5/114.15.2 monoclonal antibody; BioLegend) for Mel Juso and MutuDC cells, respectively, for 30 minutes on ice and then were washed twice in FACS buffer. GFP-positive cells were considered as “infected” and GFP-negative cells were considered as “uninfected”. Fold induction of MHC-II was calculated as the geometric mean fluorescence intensity (gMFI) of “infected cells”/gMFI of “uninfected cells”.

*Salmonella*-specific CD4^+^ T cell responses were assessed using peptide:MHC-II (p:MHCII) complexes. Flagellin_427–441_ peptide complexes were provided as phycoerythrin (PE)-conjugated tetramers by the NIH tetramer core facility and used to identify *Salmonella*-specific CD4^+^ T cells as described (29). Briefly, one million cells from the spleen or Peyer’s Patches were incubated with PE-flagellin_427–441_ at a final concentration of 25 nM for 1 hour at room temperature. Anti-PE magnetic beads (Miltenyi Biotec, Gaithersburg, MD) were added to each sample and incubated for 15 minutes on ice. Bead-bound cells were enriched as described (29) and cells in the bound fraction were stained with antibodies against CD4 (Alexa Fluor^®^ 700; RM4–5) and CD44 (Brilliant Violet 650™; IM7). The number of tetramer-specific (tetramer^+^) cells per spleen or Peyer’s Patches was calculated based on the number of tetramer^+^ cells acquired and tissue size as follows: # tetramer^+^ CD4^+^ T cells = (number of CD4^+^ PE^+^ T cells/10^6^ cells in sample) × number of total cells recovered from the tissue. All samples were acquired using a Cytek^®^ Aurora spectral flow cytometer and analyzed using SpectroFlo^®^ Software (Cytek Biosciences, Fremont, CA).

### 2.8 Sample collection and antibody quantification

One day prior to vaccination or challenge, blood was collected from each mouse to determine serum total IgG against core and O-polysaccharide (COPS) and flagella (FliC) by enzyme-linked immunosorbent assay (ELISA) as described previously (10). Animals with total IgG titers >50 ELISA units (EU) on day 83 were assessed for IgG1 and IgG2c by ELISA. Fecal pellets were collected one day before immunization and challenge to determine IgA titers by ELISA. Fecal pellets were collected and suspended in ice-cold PBS containing 0.01% sodium azide and 1% protease inhibitor cocktail (Millipore Sigma, St. Louis, MO) at a concentration of 100 mg stool/mL. Fecal suspensions were centrifuged at 5,000 *x g* for 10 min at 4°C, and the supernatants collected. All samples were stored at -80°C until analysis.

For measurement of antibody titers by ELISA, medium-binding 96-well microtiter plates (Greiner Bio-One, Monroe, NC, USA) were coated with either 100 μL/well of COPS or FliC antigens (30) in PBS at a concentration of 5 μg/mL and incubated overnight at 4 °C.

Plates were then washed with 0.05% Tween 20 in PBS (PBS-T) and blocked with 10% non-fat dry milk in PBS for 1 h at 37°C. Serum or fecal samples were diluted in 10% milk in PBS-T and added to the plate in duplicate wells. After incubation for 1 hour at 37°C, plates were washed with PBS-T and then treated with horse radish peroxidase (HRP)-conjugated goat anti-mouse IgG or IgA (Bio-Rad Laboratories, Carlsbad, CA) secondary antibody. After washing, the TMB Microwell Peroxidase Substrate system (KPL, Gaithersburg, MD, USA) was added to each well and then quenched with 1 M phosphoric acid. The optical density was measured at 450 nm with a VersaMax (Molecular Devices, San Jose, CA) microplate reader and titers were calculated by interpolation of absorbance values on a standard curve as previously described (31). Seroconversion in vaccinated mice was defined as a 4-fold increase in the antibody titer compared to the pre-immunization titer. The IgG2c/IgG1 value was calculated by dividing the IgG2c titer by IgG1 titer for each animal.

### 2.9 Opsonophagocytic uptake by macrophages

Murine J774A.1 macrophages (referred to as J774) were used for opsonophagocytic antibody activity assay (OPA) as described in (30). Twenty-four well plates were seeded with 4 x 10^5^ cells/well and incubated for 1 day. Log phase bacterial cultures of *Salmonella* Typhimurium D65 were prepared by performing a 1:1,000 dilution of an overnight culture in HY-Soy media and incubating until the OD_600_ reached 0.2. Forty-five microliters of this bacterial suspension were then incubated with 5 µL of heat-inactivated sera collected from mice before or after immunization with PBS, CVD 1926 or CVD 1926 Δ*steD* (Day 83) for 20 min at room temperature to allow for bacterial opsonization. Following incubation, 10 µL of cell suspension (2.5 x 10^4^ CFU) was added to a 24-well plate seeded with 4.5 x 10^5^ J774 cells/well (MOI of 0.27) and incubated at 37°C in a 5% CO_2_ incubator for 45 min. Cells were washed once with PBS, replaced with DMEM containing 100 µg/mL gentamicin, then incubated at 37°C in a 5% CO_2_ incubator for 1 hr. Media was removed, cells were washed three times with PBS, and lysed using 500 μL of 0.5% (wt/vol) Triton X-100. Intracellular bacteria were enumerated by viable counts. Results are expressed as fold uptake (the number of bacteria opsonized with serum, divided by the number of bacteria opsonized with no serum). Each serum sample was tested in duplicate wells at least three separate times.

### 2.10 Statistical test

Data were analyzed using GraphPad Prism 7 Software (La Jolla, CA, USA). A *p*-value equal to or below 0.05 was considered significant for each test. Mann-Whitney U test (two-tailed, α = 0.05) was used for analysis of MHC-II-fold induction, number of tetramer^+^ T cells, antibody responses, and bacterial organ burden. Fisher’s exact test (two-tailed) was used to compare seroconversion between vaccine groups.

## 3 Results

### 3.1 *SteD* deletion from wild-type *S*. Typhimurium and a live attenuated vaccine strain augments MHC-II expression

**Figure 1:**
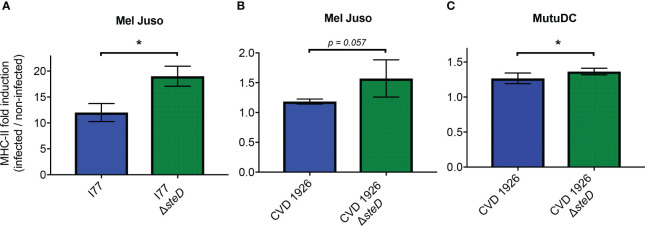
MHC-II expression in Mel Juso and MutuDC cells. **(A)** Mel Juso cells were infected with *S*. Typhimurium I77 or I77 Δ*steD.* **(B)** Mel Juso and **(C)** MutuDC cells were treated with CVD 1926 or CVD 1926 Δ*steD* for 18 hours. MHC-II surface levels were determined by flow cytometry. Fold induction refers to the increased fold change of MHC-II levels of GFP-positive cells (infected) versus GFP-negative (uninfected) cells. Results are shown as median with the range and are representative of two separate experiments. *P*-values were calculated using the Mann-Whitney test (*, *p* ≤ 0.05).

To confirm that deletion of *steD* from *S*. Typhimurium I77 and CVD 1926 increases MHC-II expression, Mel Juso and mutuDC cells were treated with their respective *steD* mutants and the surface levels of MHC-II were assessed by flow cytometry. Upon infection with GFP-expressing *S*. Typhimurium I77 or I77 Δ*steD*, Mel Juso cells infected with *S*. Typhimurium I77 Δ*steD* showed a significantly higher fold induction of MHC-II 18 hours post-infection than wild-type *S*. Typhimurium (**Figure 1A**). To determine whether deletion of *steD* from a live attenuated vaccine strain improves MHC-II expression, Mel Juso cells were infected with GFP-expressing CVD 1926 and CVD 1926 Δ*steD*. After 18 hours of treatment, the MHC-II surface levels were almost significantly higher for cells treated with CVD 1926 Δ*steD* versus CVD 1926 (**Figure 1B**; *p* = 0.057, Mann-Whitney test). To evaluate the effect of *steD* on MHC-II expression in an antigen presenting cell type, mutuDCs (dendritic cell line) were infected with GFP-expressing CVD 1926 and CVD 1926 Δ*steD*. Eighteen hours later, mutuDCs receiving CVD 1926 Δ*steD* showed significantly higher MHC-II levels as compared to CVD 1926 (**Figure 1C**).

### 3.2 CVD 1926 Δ*steD*, but not CVD 1926, induces flagellin-specific CD4^+^ T cells in aged mice following vaccination

**Figure 2:**
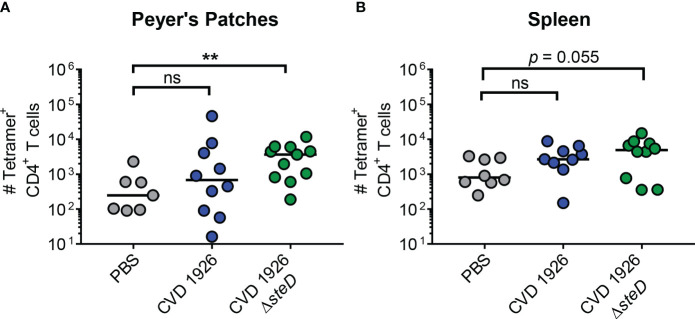
Analysis of antigen-specific CD4^+^ T cells in aged mice. C57BL/6 mice were immunized perorally with two doses of PBS, CVD 1926 or CVD 1926 Δ*steD.* Twenty-one days after the second dose, the spleen and Peyer’s Patches were removed for tetramer analysis. The number of flagellin_427–441_-specific CD4^+^ T cells in the **(A)** Peyer’s Patches and **(B)** spleen of C57BL/6 mice were determined. The bar indicates the median. *P*-values were calculated using the Mann-Whitney test (ns, not significant; **, *p* ≤ 0.01).

To determine if deletion of *steD* affects the generation of antigen-specific T cells to CVD 1926 *in vivo*, aged C57BL/6 mice were immunized with two doses of PBS, CVD 1926 or CVD 1926 Δ*steD* and the number of *Salmonella*-specific CD4^+^ T cells was measured using FliC peptide tetramers and flow cytometry. Compared to mice that received PBS, CVD 1926 Δ*steD* elicited significantly more tetramer^+^ CD4^+^ T cells in the Peyer’s Patches of aged mice (**Figure 2A**), with a 33.4-fold increase in the median number of cells as compared to PBS mice. In the spleen, the number of tetramer^+^ CD4^+^ T cells in CVD 1926 Δ*steD*-immunized mice was almost significantly different from mice that received PBS (*p* = 0.055, Mann-Whitney test), with a 2.6-fold increase in the median number of cells as compared to PBS mice (**Figure 2B**). Importantly, the number of tetramer^+^ CD4^+^ T cells in the Peyer’s patches and spleen of CVD 1926-immunized mice was not significantly different from the PBS group (**Figures 2A, B**).

### 3.3 Serum antibody response to CVD 1926 Δ*steD*

**Figure 3:**
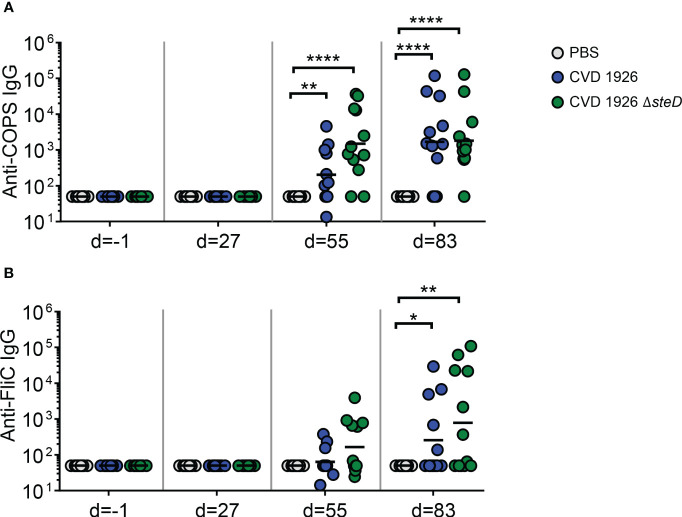
Serum IgG levels induced by CVD 1926 and CVD 1926 Δ*steD* in aged mice. **(A)** Anti-COPS and **(B)** anti-FliC serum IgG responses in mice immunized with PBS, CVD 1926, or CVD 1926 Δ*steD* at indicated time-points. Geometric mean titer represented by bar (*, *p* ≤ 0.05; **, *p* ≤ 0.01; ****, *p* ≤ 0.0001 by Mann-Whitney).

For evaluation of antibody responses to CVD 1926 Δ*steD* in aged mice, the anti-COPS and anti-FliC serum IgG titers were determined by ELISA. Aged C57BL/6 mice received three doses of PBS, CVD 1926 or CVD 1926 Δ*steD* perorally, spaced four weeks apart. Both CVD 1926 and CVD 1926 Δ*steD* induced anti-COPS IgG levels that were significantly higher than PBS-vaccinated mice after two doses (day 55) and three doses (day 83) of vaccine (**Figure 3A**). After 2 doses, most aged mice receiving CVD 1926 Δ*steD* seroconverted (10/12 [83%]) and elicited a geometric mean titer (GMT) of 1508, while less than half of the mice immunized with CVD 1926 seroconverted (5/12 [42%]) and demonstrated a GMT of 202, although the differences were not significant (**Table 2**). After 3 doses, CVD 1926 and CVD 1926 Δ*steD* induced seroconversion for serum anti-COPS IgG in 9/12 (75%) and 11/12 (92%) mice, respectively (**Table 2**). The anti-FliC responses were less robust; compared to PBS mice, there were no significant differences in serum anti-FliC levels after two doses of CVD 1926 or CVD 1926 Δ*steD*. However, 3 doses of both CVD 1926 and CVD 1926 Δ*steD* elicited significantly higher anti-FliC titers in comparison to unvaccinated mice (CVD 1926: *p* ≤ 0.05; CVD 1926 Δ*steD*: *p* ≤ 0.01) (**Figure 3B**).

**Table 2:** Serum anti-COPS and anti-FliC seroconversion and geometric mean titers (GMT).

Vaccine	Day	Anti-COPS		Anti-FliC	
		Seroconversion^a^	GMT (range)	Seroconversion^a^	GMT (range)
**CVD 1926**	56	5/12 (42%)	202 (50 - 4555)	2/12 (17%)	63 (50 - 376)
	83	9/12 (75%)	1684 (50 - 43025)	4/12 (33%)	254 (50 – 29359)
**CVD 1926 Δ*steD***	56	10/12 (83%)	1508 (50 – 36555)	5/12 (42%)	166 (50 – 3942)
	83	11/12 (92%)	1828 (50 – 42955)	6/12 (50%)	784 (50 – 108903)

a , Number of seroconverting mice/number of immunized mice (%).

**Figure 4:**
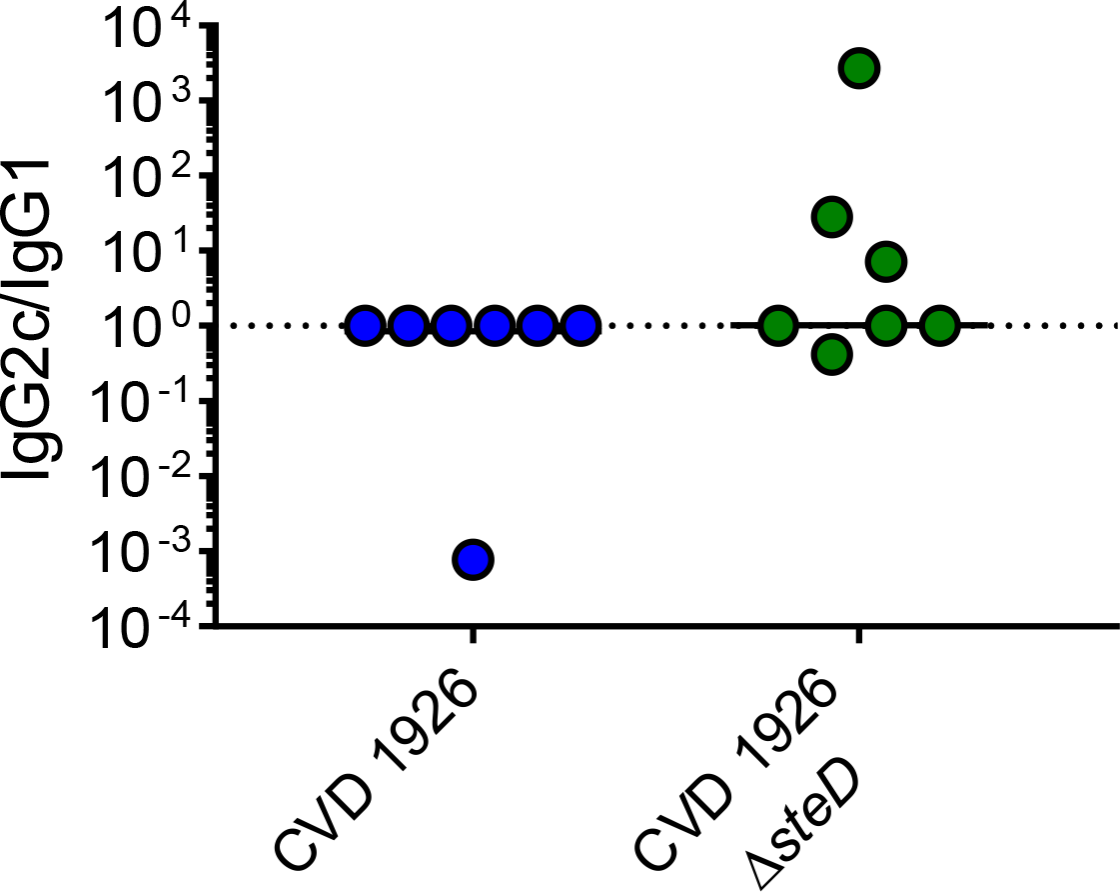
IgG isotype analysis of anti-COPS sera from mice immunized with 3 doses of CVD 1926 or CVD 1926 Δ*steD*. IgG1 and IgG2c subclasses against *S*. Typhimurium COPS were determined by ELISA on day 83. The ratio of IgG2c to IgG1 levels is reported. The bar indicates the median. *P*-value calculated using Mann-Whitney test.

Since IgG2c and IgG1 antibodies are associated with a Th1 and Th2 response, respectively, IgG isotype analysis was performed on a subset of sera from animals 1 day prior to challenge (day 83) and the ratio of IgG2c to IgG1 titer was determined (**Figure 4**). Although there was no significant difference in IgG2c/IgG1 ratio between CVD 1926- and CVD 1926 Δ*steD* -immunized mice (both had a median of 1; *p* = 0.20, Mann-Whitney test), 3/7 mice immunized with CVD 1926 Δ*steD* had an IgG2c/IgG1 ratio greater than 1, in contrast to 0/7 mice immunized with CVD 1926.

**Figure 5:**
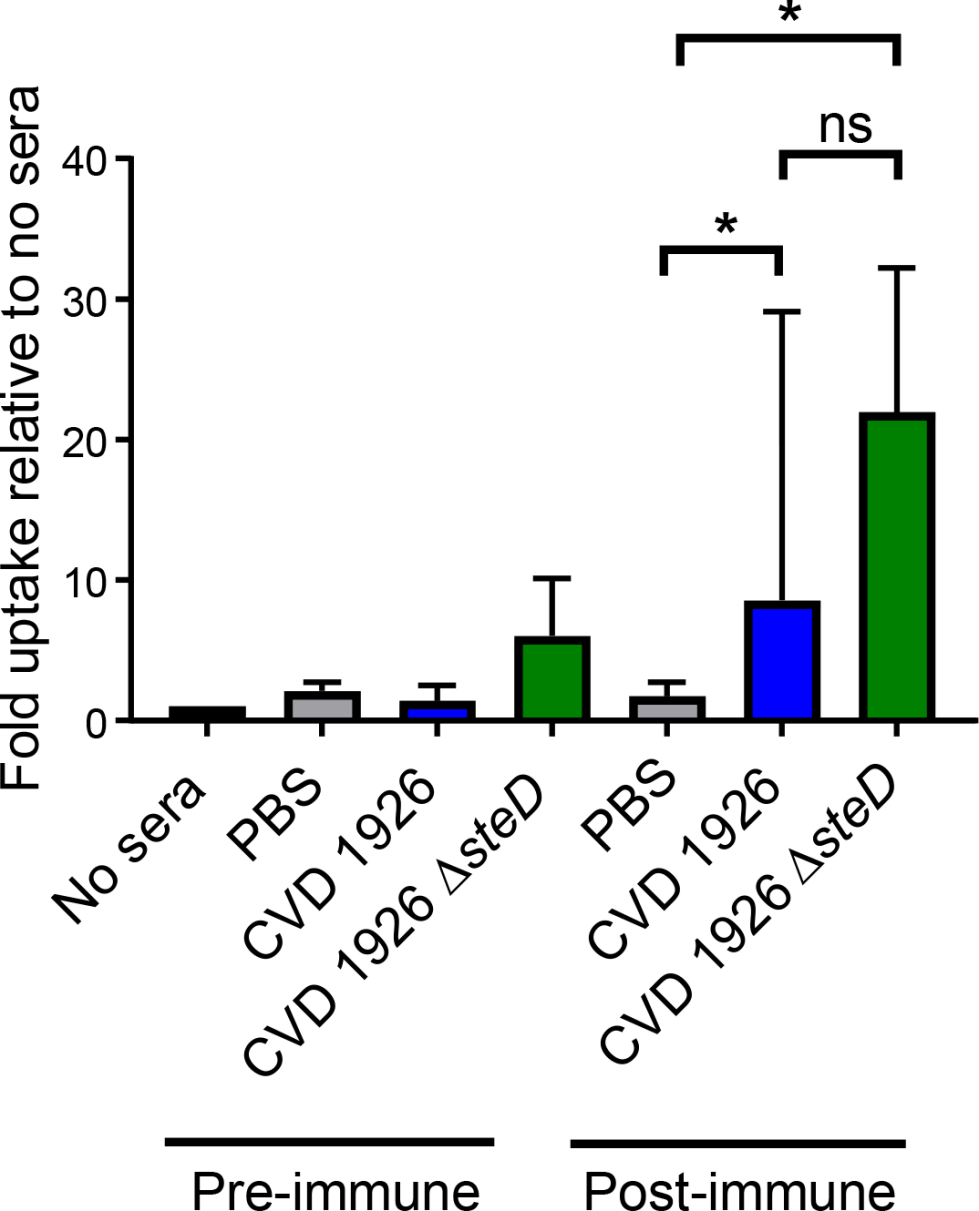
Ability of sera from CVD 1926 and CVD Δ*steD* immunized aged mice to opsonize and mediate uptake of *S.* Typhimurium by J774 murine macrophages. J774 macrophages were infected with *S*. Typhimurium opsonized with pre-immune sera or sera (Day 83) harvested from mice vaccinated with 3 doses of PBS, CVD 1926, or CVD 1926 *ΔsteD*. Results are shown as the median and the range from three independent experiments. ns, not significant; *, *p* ≤ 0.05; by Mann-Whitney.

We next assessed whether sera from immunized mice was functional and able to mediate bacterial opsonization and stimulate uptake by murine macrophages. We found that post-immune sera from CVD 1926 Δ*steD*-immunized mice were able to promote significantly higher uptake by J774 macrophages compared to post-immune PBS sera (medians of 21.95 versus 1.75, respectively; *p* ≤ 0.05, Mann-Whitney test) (**Figure 5**). Additionally, opsonization with sera from CVD 1926-immunized mice were able to promote a significant difference in uptake by J774 cells compared to sera from PBS animals (medians of 8.55 versus 1.75, respectively; *p ≤* 0.05, Mann-Whitney test). There was no significant difference in uptake between CVD 1926 and CVD 1926 Δ*steD* post-immune sera.

### 3.4 Fecal IgA response following vaccination

**Figure 6:**
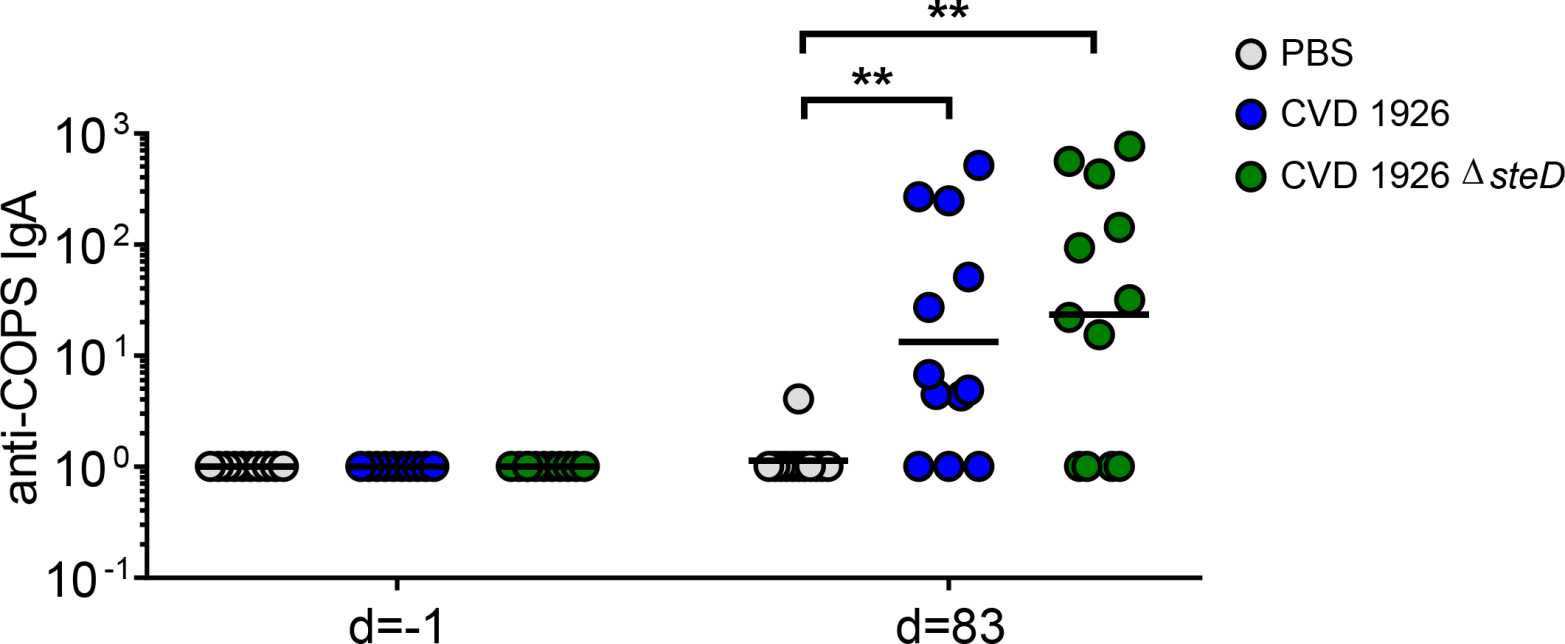
Fecal IgA responses induced by CVD 1926 and CVD 1926 Δ*steD* in aged mice. Anti-COPS IgA responses at baseline (day -1) and after 3 doses of PBS, CVD 1926, or CVD 1926 Δ*steD* (day 83). Geometric mean titer represented by bar (**, *p* ≤ 0.01 by Mann-Whitney).

For evaluation of mucosal antibody responses post-vaccination, fecal IgA levels were measured using ELISA. One day prior to challenge, both CVD 1926- and CVD 1926 Δ*steD*-immunized mice produced anti-COPS fecal IgA levels that were significantly higher than unvaccinated animals (**Figure 6**). The number of seroconverting animals for anti-COPS IgA was 9/12 (75%) and 8/12 (67%) for CVD 1926 and CVD 1926 Δ*steD*, respectively (**Table 3**).

**Table 3:** Fecal anti-COPS IgA seroconversion and geometric mean titers.

Vaccine	Day	Anti-COPS fecal IgA	
		Seroconversion	GMT (range)
**CVD 1926**	83	9/12 (75%)	13.3 (1 – 514)
**CVD 1926 Δ*steD***	83	8/12 (67%)	23.4 (1 – 765)

### 3.5 CVD 1926 Δ*steD* immunization, but not CVD 1926, reduces bacterial burden of *S*. Typhimurium in aged mice upon challenge

**Figure 7:**
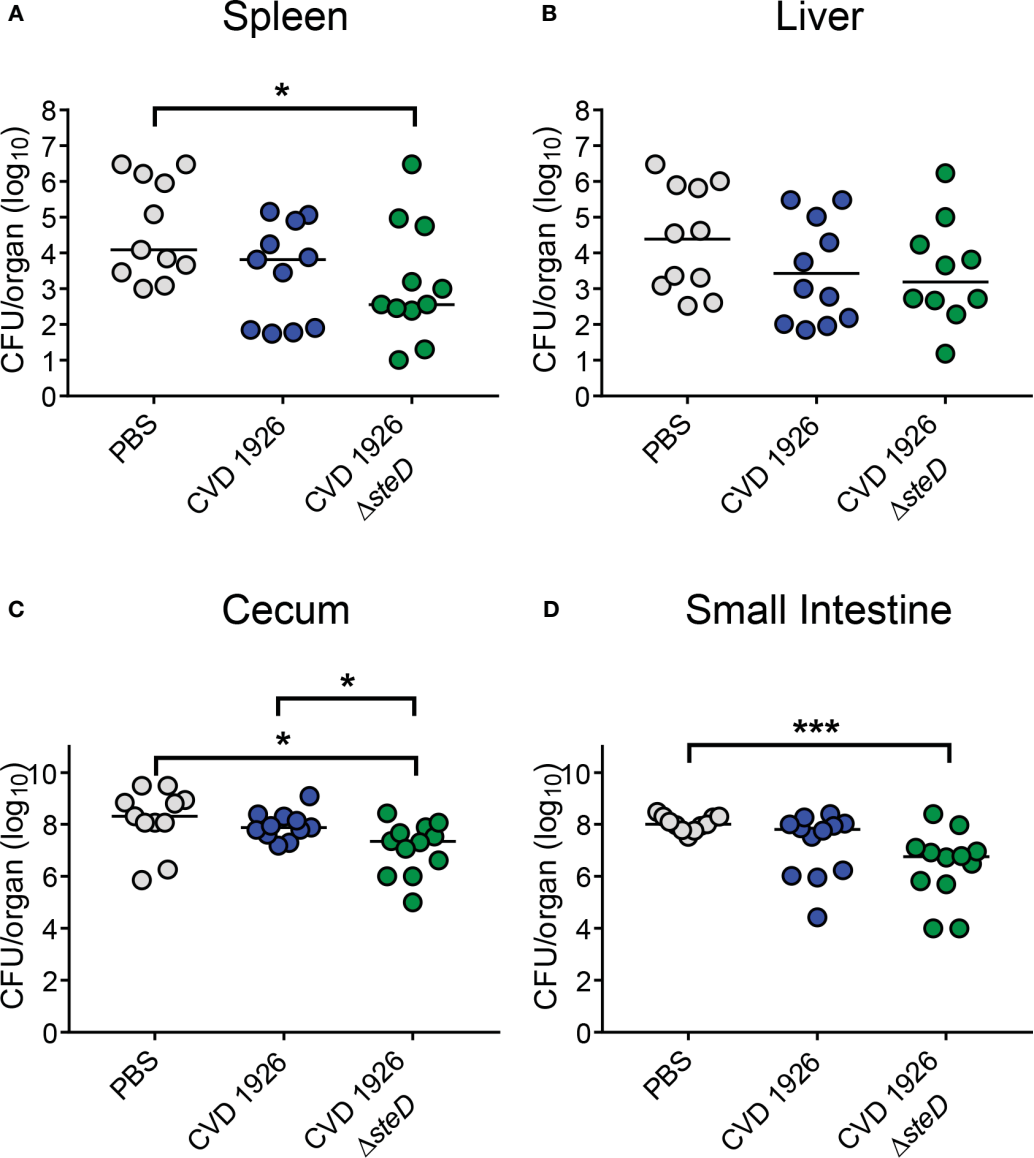
Bacterial burden of immunized aged mice after challenge with wild-type *S*. Typhimurium. Bacterial burden of the **(A)** spleen, **(B)** liver, **(C)** cecum, and **(D)** small intestine of aged C57BL/6 mice immunized with PBS, CVD 1926 or CVD 1926 Δ*steD*, and then subsequently challenged perorally with 10^8^ CFU of *S*. Typhimurium SL1344. Twenty-four hours prior to challenge, mice were pretreated with peroral streptomycin. Median represented by bar (*, *p* ≤ 0.05; ***, *p* ≤ 0.001; by Mann-Whitney).

To evaluate the effect of *steD* deletion on the ability of CVD 1926 to reduce bacterial colonization of the spleen, liver, and small intestine upon *S*. Typhimurium challenge, mice were challenged with 10^8^ CFU of *S*. Typhimurium SL1344 perorally 4 weeks after receiving 3 doses of either PBS, CVD 1926, or CVD 1926 Δ*steD*. At day 3 after challenge, CVD 1926 Δ*steD* immunization significantly reduced the CFU counts of bacteria in the spleen, cecum and small intestine compared to PBS-vaccinated mice; mice that received PBS contained 34-, 9-, and 18-fold higher median CFU counts in the spleen, cecum, and small intestine, respectively, than CVD 1926 Δ*steD*-immunized mice (**Figures 7A, C, D**). The bacterial burden of the liver was comparable between PBS- and CVD 1926 Δ*steD*-immunized mice (**Figure 7B**). Notably, there were no statistical differences in the bacterial burden of the spleen, liver, cecum nor small intestine for mice immunized with CVD 1926 versus PBS. Animals immunized with CVD 1926 Δ*steD* contained significantly less *S*. Typhimurium SL1344 in the cecum 3 days post-infection compared to animals that received CVD 1926 (*p* ≤ 0.05) (**Figure 7C**).

## 4 Discussion

While live attenuated *Salmonella* vaccines are an attractive vaccine strategy, wild-type *Salmonella* employ multiple mechanisms to subvert the host immune system, and these pathways likely remain intact in attenuated vaccine strains. Thus, we proposed that disarming a *Salmonella* immune evasion gene, *steD*, from our vaccine strain may boost antigen presentation and enhance the overall vaccine response. In this study, *steD* was deleted from CVD 1926, and we show here that *steD* mutants elicited higher MHC-II levels for multiple cell lines *in vitro*, induced higher numbers of FliC-specific CD4^+^ T cells and *Salmonella*-specific antibody responses, and reduced the bacterial burden upon challenge in aged, vaccinated mice, as compared to unvaccinated mice.

CD4^+^ T cells are critical for defense against *Salmonella* and several groups have shown that *Salmonella* specifically reduces the surface levels of MHC-II during infection, which negatively impacts CD4^+^ T cell generation (32, 33). We corroborated this finding; Mel Juso and mutuDC cells infected with *steD* mutants showed increased induction of MHC-II surface levels, as compared to the isogenic *steD^+^* strain. The increased MHC-II expression *in vitro* prompted us to evaluate if *steD* deletion from our vaccine strain could lead to an increase in *Salmonella*-specific CD4^+^ T cell levels *in vivo*. In agreement with work by David Holden’s laboratory (17, 27), incorporation of a *steD* mutation led to a higher number of FliC-specific CD4^+^ T cells in the Peyer’s Patches after two doses of CVD 1926 Δ*steD*, compared to unvaccinated mice. However, CVD 1926 did not elicit a significant increase in tetramer^+^ CD4^+^ T cells as compared to unvaccinated mice, suggesting that *steD* deletion from the vaccine strain likely improved antigen presentation and led to more antigen-specific T cells *in vivo*. Although there were 2.6-fold more tetramer^+^ CD4^+^ T cells in the spleen after CVD 1926 Δ*steD* immunization compared to unvaccinated mice, the difference was trending towards but ultimately not significant (*p* = 0.055). However, as with the response in Peyer’s Patches, CVD 1926 failed to induce FliC-specific CD4^+^ T cells in the spleen. Although migratory DCs and *Salmonella-*infected phagocytes can present antigens to splenic CD4^+^ T cells, we postulate that uptake and antigen presentation of our live oral vaccine primarily occurs in gut-associated lymphoid tissues, and this may explain why deletion of *steD* has a robust effect on Peyer’s Patch-derived T cells (34).

The effect of *steD* deletion on MHC-II expression and *Salmonella*-specific T cell numbers raises the question of whether this mutation could improve antibody responses and protection against challenge. While both CVD 1926 and CVD 1926 Δ*steD* induced high titers of anti-COPS and anti-FliC antibodies compared to PBS-immunized mice, CVD 1926 Δ*steD* elicited significantly more anti-COPS seroconverters after two doses compared to CVD 1926. Further, the GMT of anti-COPS levels of CVD 1926 Δ*steD* was 7.5-fold-higher than that of CVD 1926, suggesting that there was a difference in immunogenicity after two immunizations, but not three. This data supports the potential for vaccine dose sparing. Further, immunization with CVD 1926 Δ*steD* but not CVD 1926 resulted in fewer CFU in the spleen, cecum and small intestine compared to PBS-immunized mice following challenge. Although the anti-COPS and anti-FliC levels were comparable between vaccine groups prior to challenge (day 83), the opsonic antibody activity showed a median of 21.95 for CVD 1926 Δ*steD* and a median of 8.55 for CVD 1926, which although not significantly different, showed a large effect size and could potentially explain differences in protection. Although the difference in IgG2c/IgG1 ratio was not significantly different between vaccine groups, for some animals, CVD 1926 Δ*steD* induced predominantly IgG2c anti-COPS antibodies, which is indicative of a Th1 response, and may explain the reduction in organ burden. However, a larger sample size is necessary to determine if CVD 1926 Δ*steD* promotes IgG2c and Th1 polarization as compared to CVD 1926.

Although a complete model of how *steD* deletion enhances vaccine immunogenicity is unknown, we suspect that improved antigen presentation following vaccination results in enhanced T follicular helper cell (T_FH_) generation and/or a higher number of T helper type 1 (Th1) CD4^+^ T cells, a T cell subset required for resolution of infection. T_FH_ promote the generation of B memory and high affinity antibodies (35). However, in older adults, the T cell pool is dominated by memory T cells, rather than naïve or T_FH_ cell types, which likely contribute to the limited B cell responses to *de novo* antigens (5). A question remains whether CVD 1926 Δ*steD* elicits higher numbers of T_FH_’s following vaccination. Since CVD 1926 Δ*steD* induces antigen-specific CD4^+^ T cells, it is plausible that CVD 1926 Δ*steD-*immunized animals contain more T_FH_’s than CVD 1926-immunized mice. For example, individuals with high levels of live-attenuated influenza vaccine (LAIV)-specific T_FH_’s following immunization was associated with a robust anti-influenza antibody response (36). *Salmonella*-specific Th1 cells produce pro-inflammatory cytokines (e.g., IFN-γ, TNF- α) during infection that activate phagocytes and promote clearance of bacteria from infected tissue (37). This is supported by studies where mice lacking CD4^+^ T cell function are highly susceptible to *Salmonella* infection and convalescent typhoid fever patients produced higher levels of IFN-γ, TNF-α and MIP-1β (38–41). Therefore, a more detailed phenotypic analysis of CD4^+^ T cells following CVD 1926 Δ*steD* immunization warrants further investigation.

Although immunization with CVD 1926 Δ*steD* reduced the bacterial burden by a small amount, these results represent an improvement over aged mice that received CVD 1926. The results herein demonstrate that deletion of *steD* is a step towards the goal of an effective vaccine for older adults. *Salmonella* utilizes several immune evasion genes during infection, and we suspect that deletion of additional genes from the live vaccine strain may enable aged hosts to achieve complete protection. The results from the present study provide proof-of-concept that deletion of bacterial immune evasion genes may provide a strategy for improving the immunogenicity of live vaccines in the context of immunosenescence.

## Data availability statement

The original contributions presented in the study are included in the article. Further inquiries can be directed to the corresponding author.

## Ethics statement

Ethical approval was not required for the studies on humans in accordance with the local legislation and institutional requirements because only commercially available established cell lines were used. The animal study was approved by University of Maryland Baltimore Institutional Animal Care and Use Committee. The study was conducted in accordance with the local legislation and institutional requirements.

## Author contributions

JA: Conceptualization, Formal analysis, Investigation, Methodology, Writing – original draft. SSN: Formal analysis, Investigation, Methodology, Writing – original draft. SN: Methodology, Writing – review & editing. FT: Methodology, Writing – review & editing. ST: Conceptualization, Funding acquisition, Project administration, Supervision, Writing – review & editing.
